# Phenotypic Spectrum of Granular Corneal Dystrophy Type II in Two Italian Families Presenting an Unusual Granular Corneal Dystrophy Type I Clinical Appearance

**DOI:** 10.1155/2015/703418

**Published:** 2015-06-29

**Authors:** Cosimo Mazzotta, Claudio Traversi, Stefano Baiocchi, Stefano Barabino, Alessandro Mularoni

**Affiliations:** ^1^Ophthalmic Operative Unit, Siena University Hospital, Viale Bracci, 53100 Siena, Italy; ^2^Ophthalmic Operative Unit, Genoa University, Viale Benedetto XV, 16132 Genoa, Italy; ^3^Department of Ophthalmology, Republic of San Marino Hospital, Via La Toscana, San Marino

## Abstract

Clinical, instrumental, and genetic findings are reported in Italian families with Type II Granular Corneal Dystrophies (GCD2) presenting an initial unusual presentation of a Granular Corneal Dystrophy Type I (GCD1) phenotypic spectrum in female descendants. Slit-lamp examinations showed the typical phenotypic features of GCD2 in both mothers and a phenotypic appearance of GCD1 in both daughters. Despite the different phenotypic onset, the genetic diagnostic testing revealed the presence of a mutation in the TGFB-I gene, typical of GCD2 in both cases, excluding GCD1. Patients who were clinically suspected of corneal dystrophy need a genetic confirmatory testing for certain diagnosis. Genetic test may help to find the specific mutation distinguishing between different phenotypic spectra with relative diagnostic and prognostic implications. The study demonstrates that the phenotypic spectrum of genetically confirmed granular corneal dystrophies in patients may change over time. Since the R124H mutation has also been described in clinically asymptomatic individuals prior to LASIK, who then develop dramatic deposition, suggesting that this particular mutation and phenotype may be sensitive to, precipitated, or modified by central cornea trauma, a careful familial anamnesis excluding cornel dystrophies and specific preoperative genetic test are recommended prior to LASIK.

## 1. Introduction

Corneal dystrophies are a research field under continuous and rapid evolution. They are described as bilateral, progressive, genetically determined, and noninflammatory diseases restricted to the cornea. Several classifications have occurred over time and the 2008 IC3D classification [1] puts a strong emphasis on the importance of using genetic testing as the method for certain diagnosis.

In this classification, there are five types of corneal dystrophies including Lattice Corneal Dystrophy (LCD) Type I and variants, Granular Corneal Dystrophy Type I (GCD1), Granular Corneal Dystrophy Type II (GCD2), a.k.a. Avellino Corneal Dystrophy, Thiel-Behnke Corneal Dystrophy (TBCD), and Reis-Bückler Corneal Dystrophy (RBCD), caused by the mutation of human transforming growth factor *β*-induced (TGFB-I) gene located on chromosome 5 (genetic locus 5q31). These are categorized as the “*superfamily*” which are all autosomal dominant. TGFB-I induced protein (TGFB-I p) is a 68 kDa protein also called “*keratoepithelin*” or “*Big-h3*” that is found in the extracellular matrix of several human tissues, particularly abundant in the cornea. Mutations of TGFB-I gene encoding for TGFB-I p are associated with variable protein aggregation and deposition (amyloid and nonamyloid aggregates) in the cornea. To date at least 30 mutations of TGFB-I are known to cause corneal dystrophies. A unique property of TGFB-I p compared to other proteins linked to hereditary proteins misfolding diseases is that specific single-point mutations in TGFB-I consistently cause phenotypically different corneal dystrophies related to the nature of protein aggregates. The structural basis for the different types of TGFB-I p depositions remains unknown; however recent studies suggest that proteolytic degradation of TGFB-I p plays a fundamental role in the pathobiology of the TGFB-I induced corneal dystrophies due to a less susceptibility to proteolysis by thermolysin and trypsin. Moreover TGFB-I p structural changes alter electrostatic properties increasing aggregation propensity. The mutation of TGFB-I induces alterations in the processing and degradation patterns of TGFB-I p characterized by progressive deposition of proteinaceous material in the cornea. Karring at al. [2] demonstrated that the C-terminal of the mutant TGFB-I p (the last to be turned over in the normal human cornea) becomes more resistant to proteolysis and less degradable. Thus, increased accumulation of C-terminal mutant TGFB-I p isoforms might be the direct cause of the TGFB-I p aggregation (increased protein stability with reduced proteolysis) and crystalloid accumulation in GCD corneas.

GCD2 was discovered for the first time in 1988 by Folberg et al. [3], in families who emigrated to the United States of America from the province of Avellino in Italy. Before 1988 there was no distinction between Type I and Type II GCD. In the paper from 1988 [3] was presented the histopathology of corneal buttons from four patients, whose origins could be traced back to the Italian province of Avellino, who underwent unilateral keratoplasty because of decreased vision. These patients were clinically diagnosed as cases of GCD2. The cause was found in a mutation of TGFB-I gene, located on chromosome 5 (5q31), resulting in a base pair transition at nucleotide position 418 (guanidine to adenosine), which converts an arginine at codon 124 into a histidine [4].

Although, to date, it is not known how this mutation can lead to the disease, recently a team of South Korean researchers proposed that the pathogenesis of GCD2 has to be traced back to an impaired autophagy and to a delayed autophagy clearance of the mutant TGFB-I p [5]. The term Avellino referring to GCD2 is now considered obsolete because of the dystrophy's global occurrence. Homozygous patients have earlier onset (first decade) compared with heterozygous (second decade of life). GCD2 phenotype varies considerably according to genetics (homozygous patients demonstrate larger, deeper, and numerous deposits) and age (by adulthood there are larger, denser, and deeper deposits). Vison slowly decreases with age as the corneal central visual axis becomes progressively opaque and painful recurrent epithelial erosions may be associated. Light microscopy shows hyaline and amyloid deposits extending from the basal epithelium to the deep stroma that stain with Masson trichrome and/or Congo red.

A common ultrastructural finding between GCD2 and GCD1 is the presence of randomly aligned amyloid fibrils in the anterior stroma with more severe findings in homozygotes [6, 7].

## 2. Case Presentation

### 2.1. Case 1

**Figure 1:**
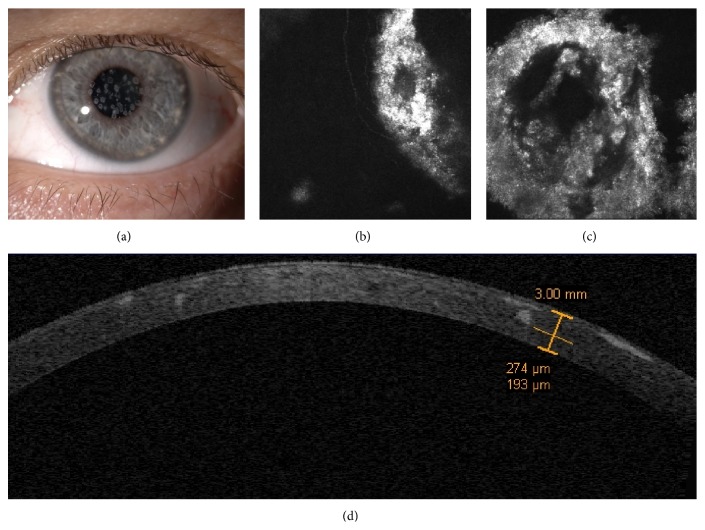
Case 1, the mother. Corneal biomicroscopy at 25x magnification shows multiple round, sharply demarcated whitish granular deposits in the central cornea. Stellate opacities, though in numerical inferiority, are also detectable (a). IVCM reveals focal multiple hyperreflective circular deposits with dense aspect (maybe of amyloid origin) surrounding internal multiple irregular hyperreflective spots (maybe of hyaline origin), often surrounding the subepithelial nerve fibers (b and c). Time domain corneal OCT scan provides an overall view of the cornea with topographic detection and depth estimation of hyperreflective deposits (d).

A 43-year-old Italian woman was referred to the University Department of Ophthalmology in Siena (Italy) with a clinical diagnosis of GCD2 (Avellino Corneal Dystrophy) who underwent a penetrating keratoplasty (PK) in her right eye 6 years before our visit. Corrected distance visual acuity (CDVA) was 20/40 in the PK eye and 20/30 in the left eye. Slit-lamp examination of the left eye revealed the presence of multiple round, sharply demarcated whitish granular deposits in the central cornea. Stellate opacities, though in numerical inferiority, were also detectable (Figure 1(a)).

Patient underwent in vivo confocal microscopy (IVCM) by the HRT II scanning laser confocal microscope (Rostock Cornea Module, Heidelberg, Germany) that showed focal multiple hyperreflective circular deposits with dense aspect (maybe of amyloid origin) surrounding internal multiple irregular hyperreflective spots (maybe of hyaline origin). Corneal deposits started at sub-Bowman level, often surrounding the subepithelial plexus nerve fibers being present in the deeper stromal layers until 250–300 *μ*m of depth (Figures 1(b) and 1(c)).

Time domain corneal OCT scan (Visante, Zeiss Meditec, Jena, Germany) provided an overview of the whole cornea allowing a topographic localization and depth measurements of the hyperreflective deposits associated with the GCD (Figure 1(d)).

**Figure 2:**
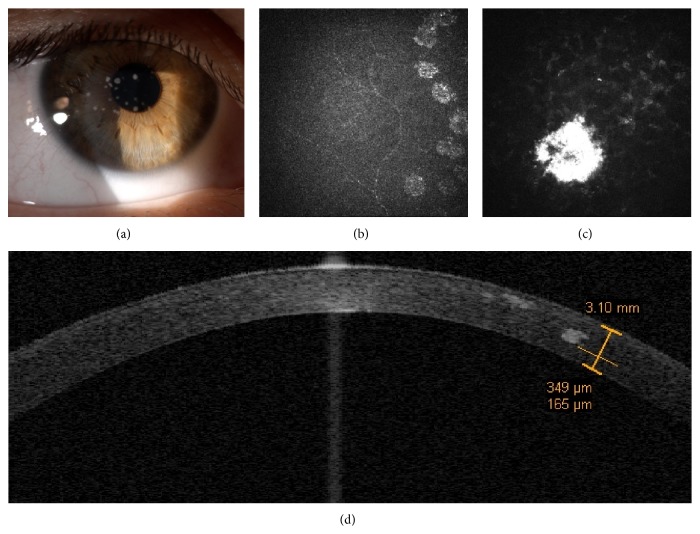
Case 1, the daughter. Slit-lamp examination shows the presence of white patchy stromal opacities, without stellate and linear opacities (a). IVCM reveals necklace-shaped opacities surrounding the subepithelial nerve fibers and deep dense granular deposits (b and c). Time domain OCT corneal scan reveals the topographic localization and the depth of corneal deposits (d).

Unlike her mother, the daughter's cornea at slit-lamp examination showed a phenotype appearance more similar to a GCD1 (Groenouw Type I) with the presence of white patchy stromal opacities, without stellate and linear ones (Figure 2(a)).

IVCM provided an insight of the deposits showing dense granular hyperreflective spots (maybe of amyloid origin) starting at sub-Bowman level until 300 *μ*m of corneal stroma (Figures 2(b) and 2(c)). In addition, time domain OCT corneal scan revealed the topographic localization and the depth of corneal deposits (Figure 2(d)).

**Figure 3:**
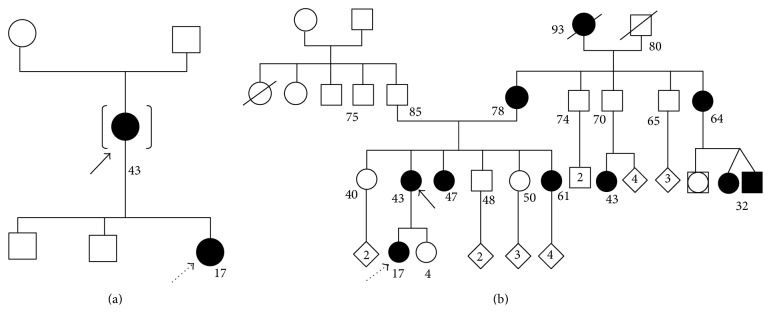
Genetic family trees of case 1 (a) and case 2 (b). The continuous black arrows indicate the mothers and the dotted black arrows indicate the daughters.

After specific consent subscription for human genetic examination, both family members underwent genetic diagnostic testing by AGDS Avellino Gene Detection System (Avellino Lab USA, Inc., Menlo Park, San Francisco, CA). After genetic testing requests including patients' clinical information, samples from the inside of both cheeks to capture sufficient cells on the collection swabs were collected. Samples were stored in refrigerator one day and sent for the analysis by courier to Avellino Lab USA, Inc., San Francisco, CA. After DNA extraction for GCD2 mutation test, the results showed that the mother was positive for GCD2, as a heterozygous carrier of the Arg124His mutation of the TGFB-I gene on chromosome 5 (genetic locus 5q31). Moreover, her daughter was found to be a carrier of the same mutation on the same gene (Figure 3(a)).

### 2.2. Case 2

**Figure 4:**
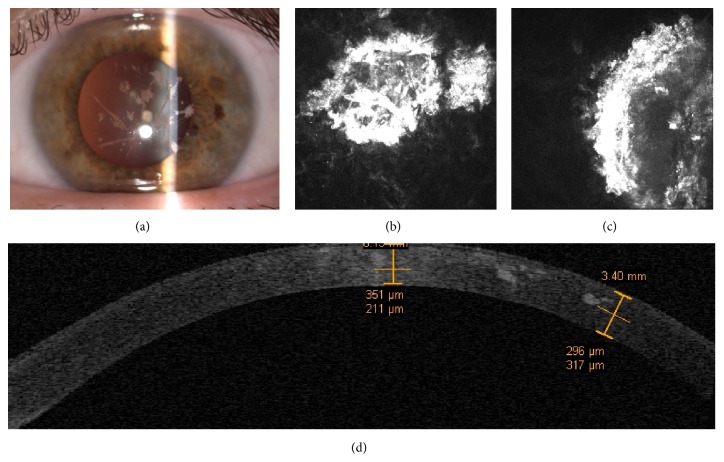
Case 2, the mother. Slit-lamp examination revealed the presence of bilateral diffuse linear or lattice, granular, and stellate opacities (a). The IVCM evaluation of the mother's cornea reveals the presence of multiple hyperreflective stellate deposits (maybe of amyloid origin) with needle-shaped dense aspects surrounding low-reflective internal core (maybe of hyaline origin) containing hyperreflective spots (b and c). Time domain OCT corneal scans provided a wide visualization of the cornea revealing the topographic localization and the depth of corneal deposits (d).

A 43-year-old mother, coming from the Italian province of Avellino, with a long familial history of corneal dystrophies, was referred to our department with a clinical diagnosis of classic Lattice Corneal Dystrophy Type 1 (LCD1). CDVA was 20/30 in the right eye and 20/40 in the left eye. Slit-lamp examination revealed the presence of bilateral diffuse linear, granular, and stellate opacities suggesting to us a clinical phenotype of GCD2 instead of classic LCD1 (Figure 4(a)).

**Figure 5:**
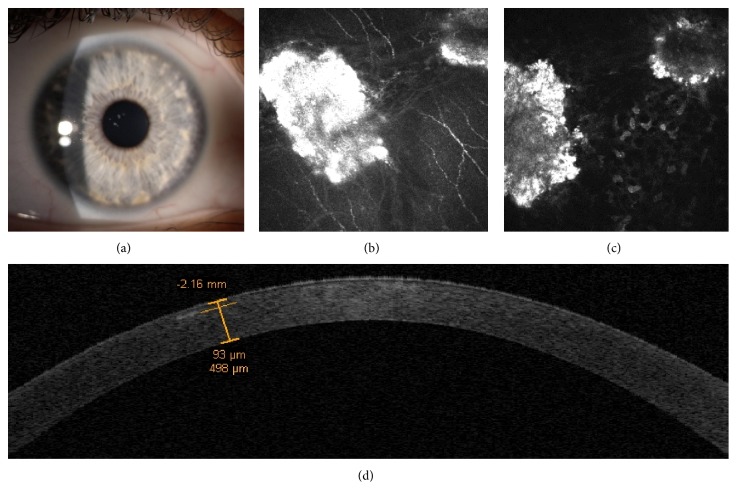
Case 2, the daughter. Slit-lamp examination shows the presence of few and very tiny patchy stromal opacities, without stellate and linear opacities at onset (a). IVCM scans show the presence of granular hyperreflective spots without complex deposits among the subepithelial nerve fibers and circular hyperreflective deposits (amyloid) surrounding an internal hyporeflective core (hyaline) in the anterior stroma (b and c). Time domain OCT corneal scans provided a wide visualization of the cornea revealing the topographic localization and the depth of corneal deposits (d).

Her 17-year-old daughter presented only few granular deposits (without linear or stellate appearance) in her right eye at biomicroscopic examination (Figure 5(a)).

IVCM evaluation of the mother's cornea revealed the presence of multiple hyperreflective circular deposits (maybe of amyloid origin) with dense aspect surrounding internal irregular hyperreflective spots (maybe of hyaline origin). Corneal deposits started at sub-Bowman level, often following the course of the subepithelial nerve fibers, being also present in the deeper stromal layers until 350 *μ*m of stromal depth (Figures 4(b) and 4(c)).

IVCM of the daughter's right eye showed the presence of granular hyperreflective spots without complex deposits starting from sub-Bowman layer under 100 *μ*m of stromal depth (Figures 5(b) and 5(c)). In addition, the OCT corneal scans provided a wide visualization of the cornea revealing the topographic localization and the depth of corneal deposits in both family members (Figures 4(d) and 5(d)).

The gene test performed by the AGDS Avellino Gene Detection System (Avellino Lab USA, Inc., Menlo Park, San Francisco, CA) stated unequivocally that both family members (the mother and the daughter) were positive for GCD2 as heterozygous carriers of the Arg124His mutation of the TGFB-I gene on chromosome 5 (Figure 3(b)).

## 3. Conclusions

This mini case series corroborates what was highlighted in the 2008 IC3D classification [1] of corneal dystrophies, as renewed in 2014 [6], demonstrating the phenomenon of phenotypic heterogeneity where an identical gene mutation may have different phenotypes. This study also demonstrated that phenotypic spectrum of corneal dystrophies may change over time with different appearance of corneal deposits at onset compared with adulthood features.

Despite their clinical phenotypes of GCD2 in both mothers and apparent phenotype of GCD1 in both daughters, all the family members examined in this case series were positive for the Arg124His mutation of the TGFB-I gene on chromosome 5, unequivocally confirming the diagnosis of GCD2 in all cases (a.k.a. Avellino Corneal Dystrophy).

The daughters' phenotypes were initially clinically suspected of GCD1 and might slightly evolve into a typical GCD2 phenotype in the future. In case 2 the mother was referred to our department with a suspected clinical diagnosis of classic LCD1 even if her clinical aspect was much more characteristic of GCD2, having typical stellate opacities mixed with the linear and granular stromal deposits. In both cases the time domain OCT corneal analysis provided a wide corneal overview allowing a topographic detection of corneal deposits with different depths measurements, very useful for diagnosis, follow-up, and surgical planning.

IVCM analysis [8, 9] provided an interesting insight in the microstructure of the deposits showing a mix of granular hyaline deposits associated with circular fusiform or linear deposits, maybe of amyloid origin, starting at sub-Bowman layers and involving the anterior-mid stroma until 300–350 *μ*m of depth. These morphological findings justified another alternative name given in the past to GCD2 as combined Granular-Lattice Corneal Dystrophy although the amyloid component is typically only apparent by histopathology and does not resemble lattice lines. However, despite the accurate morphological and clinical analysis, the genetic diagnostic testing represents the most important tool for certain diagnosis both in common and atypical evolving phenotypic spectra.

The natural history of these disorders is progression of the corneal deposition throughout life. Progression is faster in homozygous cases. The study demonstrates that the phenotypic spectrum of genetically confirmed GCD2 in patients may change over time with a transforming clinical appearance starting from GCD1 or LCD1 appearance and evolving to GCD2 in the adulthood with different prognosis and therapeutic responses among these variants.

Since the R124H mutation has also been described in clinically asymptomatic and “*dystrophy-free*” individuals prior to LASIK, who then develop deposition after LASIK [10, 11] suggesting this particular mutation and phenotype may be sensitive to/precipitated by/modified by trauma, genetic testing represents an important step especially in asymptomatic refractive surgery patients. Injury to the central cornea results in exacerbation of the GCD2 with dramatic acceleration of corneal deposition, opacities, and consequent visual loss. Hence LASIK is strongly contraindicated in GCD2 dystrophy, particularly in the homozygous patients. In heterozygous cases a PTK may be judiciously attempted only to reduce stromal haze or to postpone more definitive intervention by keratoplasty.
